# Telemedicine-supported assisted home hemodialysis in elderly and multimorbid patients: a real-world cohort study in a public healthcare system

**DOI:** 10.1007/s11255-026-05115-2

**Published:** 2026-04-11

**Authors:** Anna Zito, Paolo Ria, Maria Luisa Lefons, Vilma Martella, Silvia Matino, Giulia Fontò, Silvia Barbarini, Tiziana Rollo, Marcello Napoli, Antonio De Pascalis

**Affiliations:** 1 Department of Nephrology, Dialysis and Transplantation, Vito Fazzi Hospital, ASL Lecce, Lecce, Italy; 2 Department of Biological Sciences and Environmental Technologies, University of Salento, Lecce, Italy

**Keywords:** Home hemodialysis, Telemedicine, Renal dialysis, Aging population, Frailty, Health care costs, Delivery of health care, Public health systems

## Abstract

**Purpose:**

The aging of the hemodialysis (HD) population and the growing burden of multimorbidity increasingly challenge the sustainability and equity of conventional in-center dialysis within publicly funded healthcare systems. Although home hemodialysis (HHD) may offer clinical and organizational advantages, its implementation remains limited among elderly and functionally dependent patients. We evaluated the real-world feasibility, retention, and system-level impact of a telemedicine-supported assisted-HHD (A-HHD) program aimed at broadening access to complex individuals.

We performed a retrospective, descriptive observational cohort study including all patients enrolled in an A-HHD program within a public healthcare authority in Southern Italy from June 2018 to June 2025. In September 2023, a structured telemedicine platform enabling real-time remote monitoring and audiovisual supervision was integrated into routine care. We assessed epidemiological trends, patient complexity, vascular access patterns, dialysis prescription and delivered dose, treatment retention, causes of discontinuation, and transportation-related economic outcomes.

**Results:**

A total of 146 patients (mean age 79 ± 7 years) with substantial cardiovascular, metabolic, and respiratory comorbidity were treated. Central venous catheters were used in 64% of cases. Following early program expansion, annual incidence stabilized at approximately 20–25 patients, while prevalence steadily increased, exceeding 20 active patients per month. More than half of patients remained on therapy beyond 6 months. Despite shorter individual sessions, increased treatment frequency achieved a weekly standard Kt/V comparable to conventional in-center HD. Treatment discontinuation was predominantly related to mortality or clinical indications rather than program failure.

**Conclusion:**

Telemedicine-supported A-HHD represents a feasible and organizationally sustainable model capable of expanding equitable access to home-based dialysis while supporting organizational sustainability in public healthcare systems.

## Introduction

The progressive aging of the dialysis population and the increasing burden of frailty and multimorbidity are placing substantial strain on hospital-based HD services, particularly within publicly funded healthcare systems [1–3]. Despite representing a relatively small proportion of the general population, patients receiving dialysis account for a disproportionate share of healthcare expenditure, driven by treatment intensity, infrastructure requirements, and transportation needs [1, 2, 4]. In Italy, approximately 50,000 patients were receiving dialysis in 2014, with prevalence increasing markedly with age, reflecting demographic trends similar to those observed across Europe and North America [3, 5].

In this context, HHD has been proposed as a clinically safe and potentially more sustainable alternative to conventional in-center HD [6–8]. However, despite demonstrated feasibility and potential benefits, the diffusion of HHD remains limited and uneven across healthcare systems, particularly among elderly and highly comorbid patients who might benefit the most [7, 9–11]. HHD represents less than 2% of dialysis modalities in most healthcare systems, reflecting persistent barriers to implementation rather than a lack of technological capability [9, 10].

Key barriers to HHD adoption include the absence of an available caregiver, caregiver burden, cognitive impairment, physical disability, and concerns regarding self-cannulation and treatment safety [11–13]. A-HHD, supported by trained personal support workers (PSWs), has, therefore, been proposed as a strategy to overcome these barriers and extend access to home-based therapy to frail and dependent patients [12–15]. Previous studies suggest that assisted dialysis models can be acceptable and well tolerated in elderly populations, while preserving the potential organizational advantages of home-based care [13–15].

Telemedicine and remote monitoring technologies may further enhance the feasibility and scalability of A-HHD by enabling continuous clinical oversight, timely intervention, and integration of home-treated patients into routine nephrology workflows [16–19]. However, real-world evidence on the long-term clinical feasibility, organizational sustainability, and system-level impact of telemedicine-supported A-HHD within public healthcare systems remains limited, with most reports confined to pilot studies or selected populations [17–20].

Beyond clinical and organizational considerations, dialysis care carries substantial economic and environmental implications. Patient transportation represents a major contributor to healthcare costs and carbon emissions associated with dialysis delivery, particularly among elderly patients requiring assisted or non-emergency medical transport [1, 2, 21, 22]. Home-based and technology-enabled dialysis models may mitigate these impacts, but to date, long-term real-world evidence on telemedicine-supported assisted HHD in elderly and highly comorbid populations within public healthcare systems remains scarce [21, 22].

In light of these considerations, our Department of Nephrology and Dialysis initiated an assisted-HHD program supported by PSWs in 2018 and implemented a dedicated telemedicine platform in 2023. The aim of the present study was to evaluate the real-world implementation of a telemedicine-supported A-HHD program within a public healthcare system, focusing on epidemiological trends, patient complexity, organizational sustainability, and treatment delivery in an elderly and clinically complex population.

## Methods

### Study design and settings

We conducted a retrospective observational cohort study including all patients enrolled in an A-HHD program within the Local Health Authority of Lecce (Puglia, Southern Italy) between June 1, 2018 and June 30, 2025. The program operates within a public healthcare system using a hub-and-spoke model connecting the central Nephrology Unit with patients’ homes and satellite facilities. The study was exploratory and primarily descriptive; no contemporaneous control group was prespecified.

A structured telemedicine (TD) platform enabling real-time remote monitoring and audiovisual supervision was integrated into routine care in September 2023.

### Participants

All consecutive adult patients initiating A-HHD during the study period were included. There were no predefined exclusion criteria beyond standard clinical contraindications to home hemodialysis as determined by the treating nephrologist. A formal comorbidity score (for example, Charlson Comorbidity Index) was not systematically available in the clinical database; patient complexity was, therefore, described through the recorded burden of individual comorbid conditions.

Eligibility for A-HHD was based on: (i) need for maintenance hemodialysis, (ii) clinical unsuitability for autonomous HHD, (iii) presence of functional limitations, caregiver absence, or frailty, and (iv) feasibility of home installation and logistical requirements.

Patients were followed from A-HHD initiation until treatment discontinuation, death, transfer to in-center HD, kidney transplantation, or end of study period.

### The A-HHD program

Dialysis was delivered using the NxStage System One platform with individualized prescriptions. All patients received support from trained personal support workers (PSWs). PSWs were specifically trained by the nephrology team for home-dialysis logistics, machine preparation and disassembly, basic intradialytic monitoring, recognition of warning signs, and activation of the escalation pathway. Their level of autonomy was predefined according to the patient’s functional status and the vascular access in use. Routine sessions were not nurse-attended in person, but nursing and medical staff remained immediately available for remote support and for on-site reassessment whenever clinically required.

Dialysis frequency and duration were tailored to clinical needs, with regimens ranging from three to five sessions per week.

### Telemedicine integration

Telemedicine was introduced in September 2023 and applied to all patients actively receiving A-HHD at that time, while patients enrolled before that date had been managed within the pre-existing assisted home-dialysis model without digital remote supervision.

The telemedicine system included a home-based remote station, a centralized control room within the Nephrology Unit, synchronous audiovisual connectivity, and continuous transmission of treatment and vital parameters during dialysis. Monitoring was, therefore, based on real-time supervision during the treatment session, while transmitted treatment data could also be reviewed by the nephrology team within the same clinical workflow. Alerts generated during treatment were reviewed in the centralized control room by the supervising nephrology team, enabling prompt intervention when needed.

No major safety signals clearly attributable to telemedicine implementation emerged during routine clinical use; occasional technical connectivity issues were managed within the standard care workflow without permanent program interruption.

### Outcomes

The primary objective was to evaluate the real-world feasibility and sustainability of telemedicine-supported A-HHD.

Primary outcomes included:

- Annual incidence and program prevalence
- Treatment retention duration
- Causes of treatment discontinuation.

Secondary outcomes included:

- Patient demographics and comorbidity burden
- Vascular access patterns
- Dialysis prescription characteristics
- Delivered dialysis dose (single-pool Kt/V and standard Kt/V).

Dialysis adequacy was assessed using single-pool Kt/V (spKt/V) and standard Kt/V (stdKt/V) derived from routine pre- and post-dialysis urea measurements and standard urea-based formulas. For in-center HD, stdKt/V was estimated assuming a conventional three-times-per-week schedule. In this elderly and clinically complex cohort, dialysis prescriptions were individualized according to clinical tolerance and overall treatment goals rather than being driven solely by a rigid uniform adequacy target.

### Statistical analysis

Given the observational design, analyses were primarily descriptive. Continuous variables are presented as mean ± standard deviation (SD) when dispersion data were available; otherwise, descriptive program-level summary values are reported. Categorical variables are expressed as absolute numbers and percentages.

No formal inferential comparisons were planned. Trends in incidence and prevalence were evaluated descriptively over time, including the period before and after telemedicine integration. Because telemedicine was introduced late in the observation period, no formal pre/post-effectiveness analysis was prespecified.

### Ethical considerations

The study was conducted in accordance with the principles of the Declaration of Helsinki. As a retrospective analysis of routinely collected clinical data, formal informed consent was waived according to institutional policy. Patient data were anonymized prior to analysis.

## Results

The main clinical, organizational, and treatment-related results are summarized below.

### Study population and baseline characteristics

Between June 2018 and June 2025, a total of 146 patients were enrolled in the A-HHD program of the Local Health Authority of Lecce. Given the observational and program-level nature of the analysis, we did not perform a patient-level comparative stratification according to pre-telemedicine and post-telemedicine exposure. The cohort included 82 men (56.2%) and 64 women (43.8%), with a mean age at treatment initiation of 79.1 ± 7 years (range 42.6–99 years), reflecting a predominantly elderly and clinically fragile population. Patients exhibited a high burden of comorbid conditions (Table 1). Hypertension was the most prevalent comorbidity (46%), followed by diabetes mellitus (32%), respiratory diseases (21%), congestive heart failure (20%), ischemic heart disease, and malignancies (17%). Neurological disorders, cognitive impairment, and other disabling conditions were also present, contributing to the overall clinical complexity. Most patients had multiple concurrent comorbidities.

**Table 1:** Baseline demographic and clinical characteristics of patients enrolled in the assisted home hemodialysis (A-HHD) program (*N* = 146)

Variable	Value
Age (years), mean ± SD	79 ± 7
Male sex, *n* (%)	82 (56.2%)
Female sex, *n* (%)	64 (43.8%)
Vascular access: AVF, *n* (%)	51 (35%)
Vascular access: CVC, *n* (%)	95 (64%)
Obesity (BMI > 30), *n* (%)	15 (10%)
Diabetes mellitus, *n* (%)	47 (32%)
Hypertension, *n* (%)	67 (46%)
Pulmonary diseases (total), *n* (%)	31 (21%)
Chronic obstructive pulmonary disease	19
Chronic respiratory insufficiency	25
Cardiac diseases (total), *n* (%)	50 (34%)
Chronic ischemic cardiopathy	16
Chronic cardiac insufficiency	29
Atrial fibrillation	14
Valvulopathies	9
Peripheral vasculopathy, *n* (%)	4 (3%)
Dementia, *n* (%)	4 (3%)
Cerebral ischemic disease, *n* (%)	9 (6%)
Hepatopathy, *n* (%)	5 (3%)
Urinary/bowel diversion, *n* (%)	8 (5%)
Immunological disorders, *n* (%)	4 (3%)
Mental diseases, *n* (%)	6 (4%)
Neoplasms, *n* (%)	25 (17%)
Immobilization syndrome, *n* (%)	5 (3%)
Motility disorders, *n* (%)	8 (5%)
Other disabilities, *n* (%)	5 (3%)

Continuous variables are presented as mean ± standard deviation (SD) and categorical variables as number (percentage). Age is reported as mean ± SD, whereas several dialysis comparator variables were available only as aggregated program-level values. Subcategories of pulmonary and cardiac disease are not mutually exclusive

*AVF* arteriovenous fistula, *CVC* central venous catheter, *BMI* body mass index

### Vascular access and treatment assistance

**Fig. 1:**
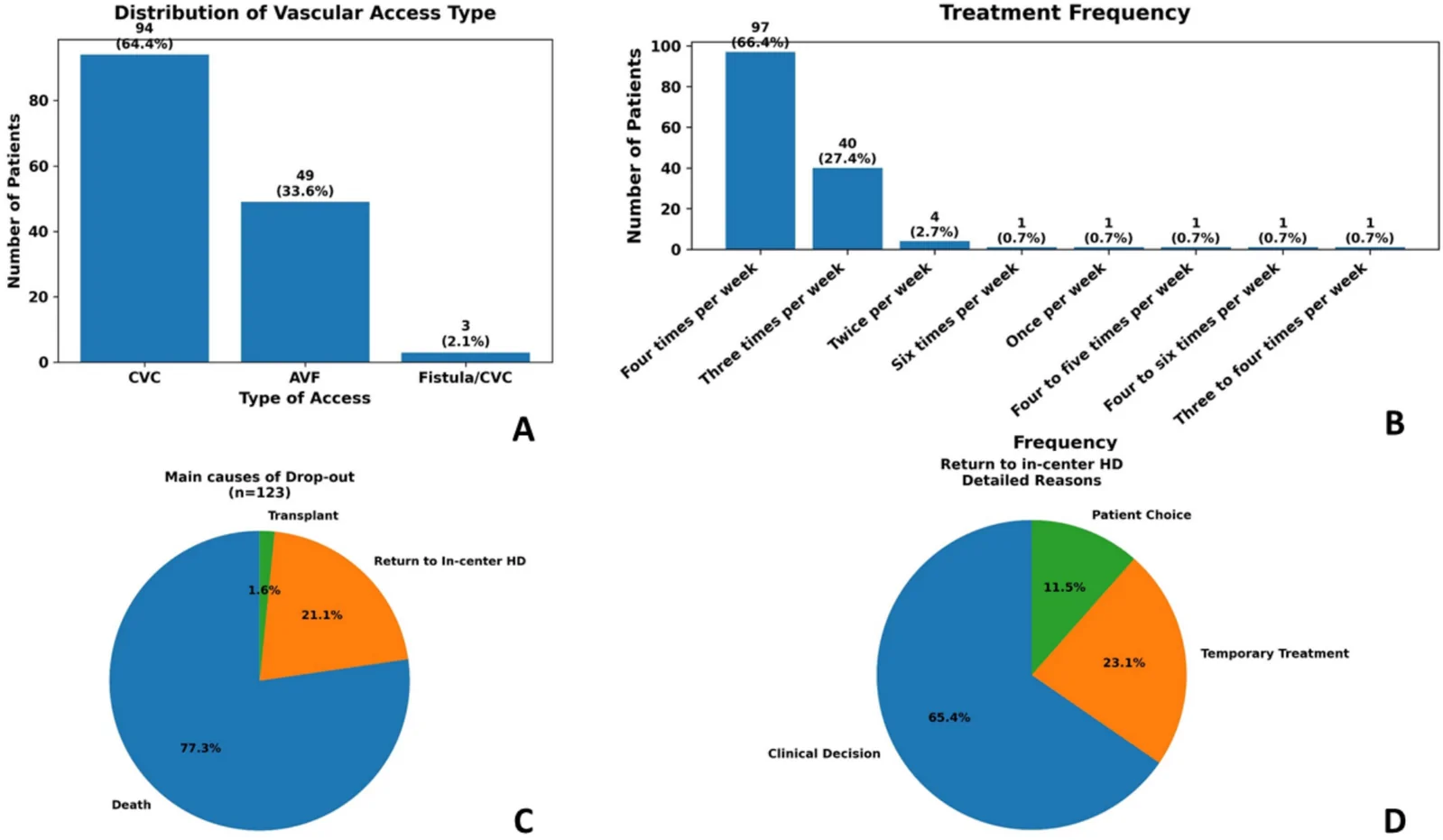
Distribution of vascular access type (**A**), dialysis treatment frequency (**B**), main causes of treatment discontinuation (**C**), and detailed reasons for temporary return to in-center hemodialysis (HD) among patients undergoing assisted home hemodialysis (A-HHD). *AVF* arteriovenous fistula, *CVC* central venous catheter, *A-HHD* assisted home hemodialysis, *TD* telemedicine

Arteriovenous fistulas (AVFs) were present in 51 patients (35%), whereas central venous catheters (CVCs) were present in 95 (64%), with 3 patients temporarily using both access types during transitional phases (Fig. 1A). All patients received dialysis with the support of trained PSWs, with the level of assistance tailored to individual functional and clinical needs. Assistance included machine set-up and disassembly, support for vascular access management according to the predefined care plan, and intradialytic monitoring under nephrology supervision.

### Dialysis prescription and delivered dose

HHD treatments were characterized by flexible dialysis prescriptions. The most frequently adopted regimen was a four-times-per-week schedule, used in approximately two-thirds of patients, followed by three- and five-times-per-week schedules (Fig. 1B). Treatment duration generally ranged from 240 to 300 min per session, according to prescription and clinical tolerance.

When compared with conventional in-center HD, which was delivered three times per week with 240-min sessions, HHD involved shorter individual treatments but higher frequency. Single-pool Kt/V was derived from routine pre- and post-dialysis urea measurements, and standard Kt/V was calculated from treatment frequency and sessional clearance using established urea kinetic methods. As expected, single-pool Kt/V per session was lower in home HD. However, when dialysis dose was expressed as standard Kt/V, HHD achieved overall weekly clearance values comparable to those estimated for conventional in-center HD (Table 2). These findings should be interpreted in the context of individualized prescriptions designed for tolerability in an elderly and highly comorbid population. Within this clinical framework, adequacy targets were approached through individualized, frequency-adjusted prescriptions rather than rigid adherence to conventional per-session thresholds.

**Table 2:** Comparison of dialysis treatment characteristics and delivered dose between conventional in-center hemodialysis and assisted home hemodialysis (A-HHD)

Parameter	In-center HD (Lecce)	Home HD (NxStage)
Treatment duration, min	240	240–300
Dialysis sessions per week	3	3.5
Pre-dialysis urea (mg/dL)	136.8	117.4
Post-dialysis urea (mg/dL)	40.6	52.6
Single-pool Kt/V (spKt/V)	1.47	0.91
Standard Kt/V (stdKt/V)	2.1 (estimated)	1.92

Values are reported as program-level mean values; measures of dispersion were not uniformly available for all comparator parameters. Standard Kt/V for in-center hemodialysis was estimated assuming a conventional three-times-per-week schedule

*HD* hemodialysis; *spKt/V* single-pool Kt/V; *stdKt/V* standard Kt/V

### Treatment discontinuation

Treatment discontinuation was primarily driven by mortality and clinical decisions, including transition to in-center HD for medical reasons. Voluntary discontinuation accounted for a small proportion of dropouts. Temporary readmissions to in-center HD occurred in selected cases and were generally related to acute clinical issues, with subsequent return to home treatment when feasible (Fig. 1C, D).

### Incidence, prevalence, and treatment retention

**Fig. 2:**
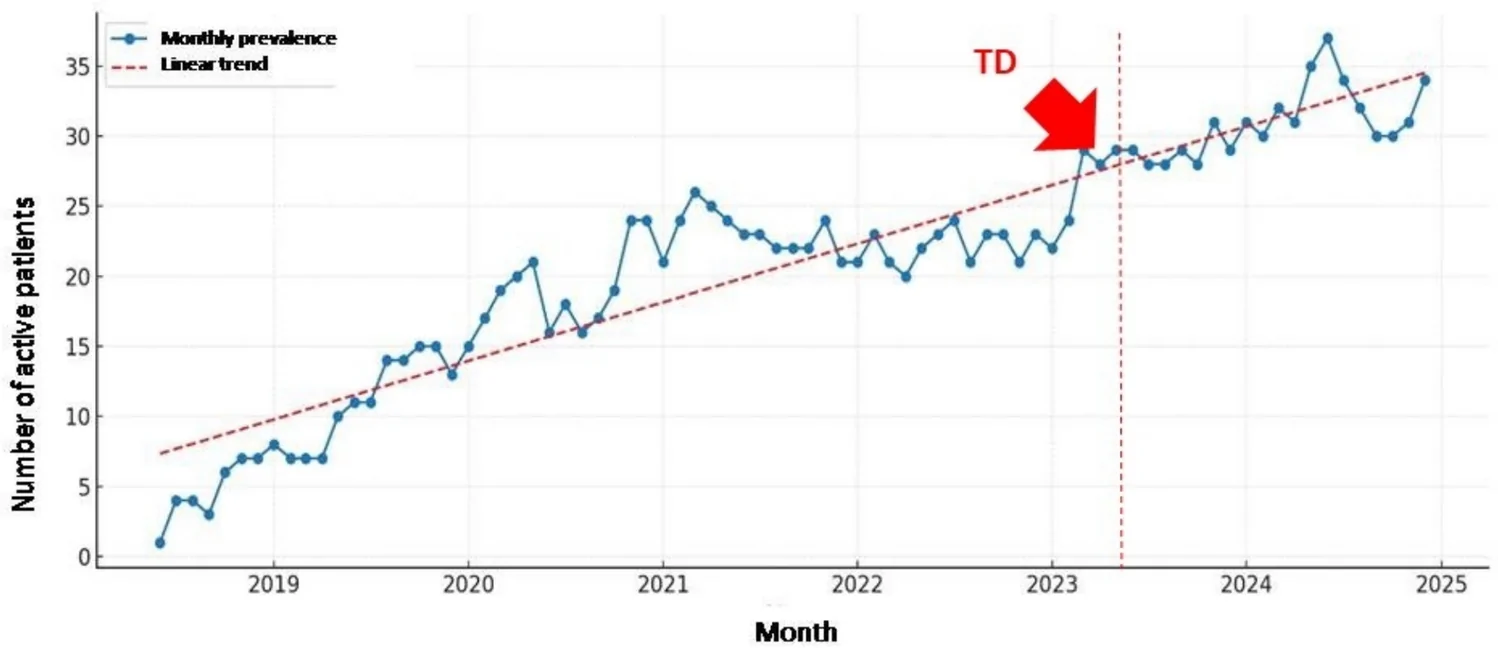
Monthly prevalence of patients receiving assisted home hemodialysis (A-HHD) from June 2018 to December 2024. The dashed vertical line indicates implementation of the telemedicine (TD) platform in September 2023. The solid line represents the linear trend. *A-HHD* assisted home hemodialysis, *TD* telemedicine

**Fig. 3:**
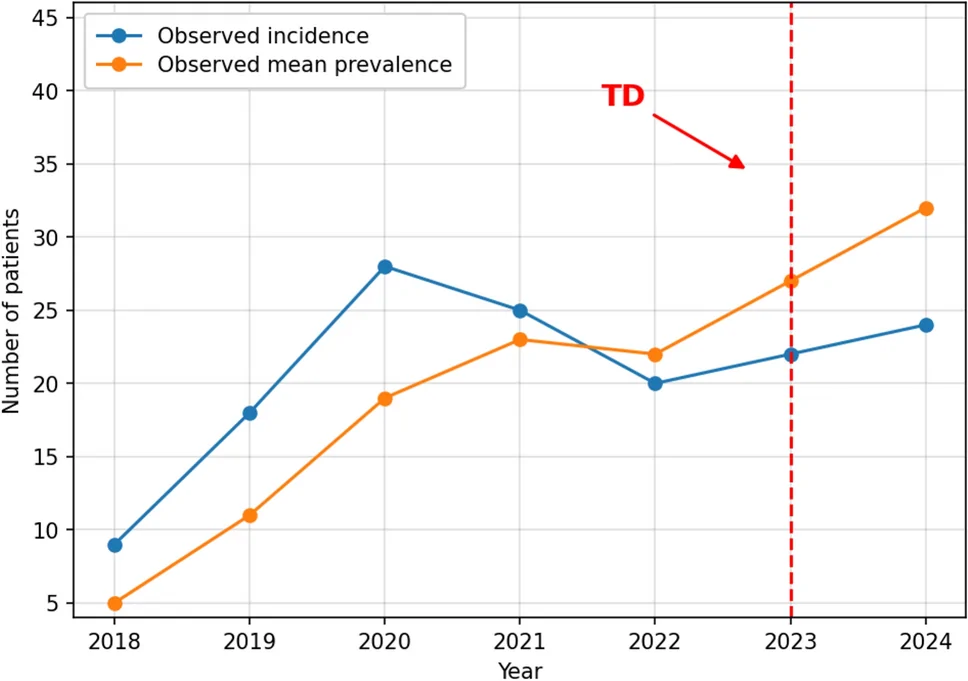
Annual incidence and prevalence of assisted home hemodialysis (A-HHD) between 2018 and 2024. The vertical dotted line marks the initiation of telemedicine integration in 2023. *A-HHD* assisted home hemodialysis, *TD* telemedicine

Annual incidence of A-HHD increased rapidly during the early phase of program implementation, rising from 9 patients in 2018 to 28 patients in 2020. In subsequent years, incidence stabilized at approximately 20–25 new patients per year (Table 3). Program prevalence increased progressively over time, reaching a stable plateau of approximately 22–24 active patients per month in the final years of observation (Figs. 2, 3). More than 50% of patients remained on A-HHD for longer than 6 months, indicating satisfactory treatment retention in this elderly and clinically complex cohort. Because telemedicine was introduced late in the observation period, Figs. 2 and 3 should be interpreted as descriptive representations of program evolution rather than as formal evidence of a telemedicine-related change in growth slope.

**Table 3:** Annual number and percentage distribution of new assisted home hemodialysis (A-HHD) enrolments between 2018 and 2024

Year	New HHD enrolments	Percentage of total (%)
2018*	9 (from June)	6.2
2019	18	12.3
2020	28	19.2
2021	25	17.1
2022	20	13.7
2023	22	15.1
2024	24	16.4

*Data for 2018 refer to the period June–December

### Economic outcomes

**Fig. 4:**
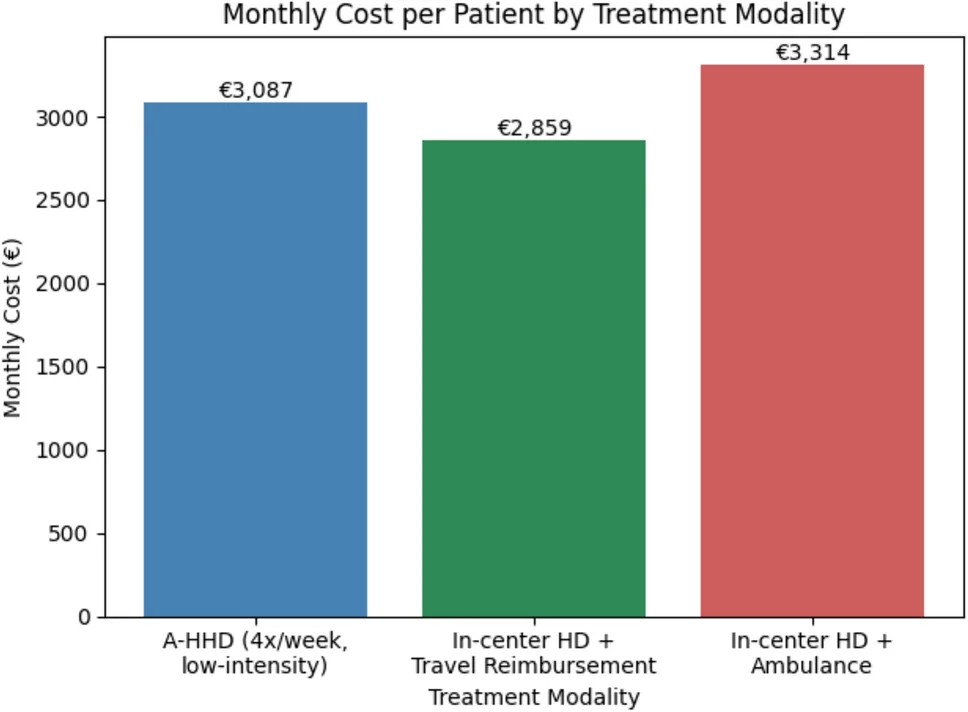
Comparison of estimated monthly transportation-related costs per patient according to dialysis modality and type of transport. Costs reflect publicly reimbursed transportation expenses within the Local Health Authority and include standard and assisted/ambulance transport. *A-HHD* assisted home hemodialysis, *TD* telemedicine

Compared with conventional in-center HD, telemedicine-supported A-HHD was associated with substantial reductions in transportation-related costs, particularly for elderly patients requiring assisted or non-emergency medical transport. Cost estimates were based on publicly reimbursed transportation tariffs within the Local Health Authority and should be interpreted as scenario-based organizational estimates rather than as a full cost-effectiveness analysis. In the scenario requiring ambulance transport, telemedicine-supported A-HHD was associated with lower transportation-related costs than in-center HD (€3,087 vs €3,314 per patient per month), corresponding to an estimated reduction of approximately €227 per month (≈7%), or €2,724 per patient annually. Not all patients treated in-center would necessarily require ambulance transport, and telemedicine equipment, infrastructure, and staffing costs were not included in this analysis. Cost savings were most pronounced in scenarios involving ambulance transportation (Fig. 4).

## Discussion

In this real-world cohort of elderly and multimorbid patients, telemedicine-supported A-HHD was feasible, sustained over time, and associated with stable program growth and satisfactory treatment retention within a public healthcare system. Our results address an important gap in the literature, where evidence on telemedicine-supported A-HHD remains largely limited to pilot studies or selected patient populations [17–20]. The descriptive nature of the study should be kept in mind when interpreting these findings.

Telemedicine was introduced in September 2023 and applied to all patients actively receiving A-HHD at that time, while patients enrolled before that date had been managed within the pre-existing assisted home-dialysis model without digital remote supervision. Because this was not a prespecified comparative analysis, outcomes were not formally stratified according to the pre- versus post-telemedicine period. However, telemedicine modified the operational workflow by enabling real-time audiovisual supervision, centralized review of treatment data and alerts, and earlier remote clinical assessment during session set-up, intradialytic monitoring, and treatment completion.

A key finding of this study concerns dialysis dose delivery. Although individual treatment sessions were shorter in HHD compared with the conventional in-center HD, increased treatment frequency resulted in comparable overall weekly solute clearance when dialysis adequacy was assessed using standard Kt/V. This observation reinforces the limitations of session-based adequacy metrics, such as single-pool Kt/V, when applied to non-conventional dialysis schedules and supports the use of frequency-adjusted measures in elderly and clinically complex patients [23–26]. In this context, shorter and more frequent treatments may represent a physiologically favorable approach, potentially improving tolerability without compromising dialysis adequacy. In our cohort, prescriptions were individualized according to hemodynamic tolerance, frailty, and clinical goals rather than being based exclusively on a single numeric adequacy threshold.

Vascular access patterns in our cohort were characterized by a predominance of central venous catheters, reflecting urgent dialysis initiation and limited vascular capital commonly observed in very old and multimorbid patients. While this finding differs from traditional fistula-first strategies, contemporary guidelines emphasize individualized, patient-centered vascular access selection, particularly in populations with limited life expectancy or high comorbidity burden [27, 28]. The presence of a substantial subgroup of patients with arteriovenous fistulas indicates that access planning remains feasible in selected candidates undergoing A-HHD. In the home setting, this pattern underscores the importance of strict catheter-care protocols, dedicated caregiver training, and rapid access to nephrology support whenever infection-related concerns arise.

Telemedicine played a central enabling role in the implementation of the program. Beyond enhancing treatment safety through remote monitoring and audiovisual supervision, telemedicine facilitated integration of home-treated patients into routine nephrology workflows [16–20, 29]. In our program, the main impact of telemedicine was organizational: it centralized remote supervision, supported real-time escalation in the presence of alerts, and strengthened the linkage between the home setting and the nephrology unit. Because the post-implementation observation window was relatively short and the study was not designed for formal comparative analyses, we report this experience descriptively rather than as a before-versus-after effectiveness evaluation. These findings align with emerging evidence on the role of digital health technologies in dialysis care, particularly following the accelerated adoption of telemedicine during the COVID-19 pandemic [18, 19, 30].

In our experience, the main contribution of telemedicine was organizational rather than experimental: it strengthened surveillance capacity, facilitated integration of home-treated patients into routine nephrology workflows, and supported clinical decision-making without changing the descriptive nature of the present study.

In addition to clinical and organizational considerations, our study highlights relevant economic and environmental implications. Dialysis-related transportation represents a major contributor to healthcare costs and greenhouse gas emissions [1, 2, 21, 22]. The economic benefit was particularly evident in patients requiring ambulance transport, where A-HHD was associated with an estimated reduction of approximately €2,700 per patient per year compared with in-center HD. Assuming a stable prevalence of 22–24 active patients per month, this would translate into an estimated annual system-level saving of approximately €60,000–65,000 in scenarios where ambulance transport would otherwise be required. These estimates should be interpreted within the boundaries of the analysis, which focused on transportation tariffs and did not incorporate telemedicine implementation costs. As we previously reported, following telemedicine integration in 2023, A-HHD was associated with lower transportation-related costs and environmental burden and a more sustainable model of renal care, particularly among elderly patients requiring assisted or non-emergency medical transport [22].

Several limitations should be acknowledged. The retrospective, single-center design limits generalizability and precludes causal inference, and the absence of a contemporaneous comparator group restricts direct effectiveness comparisons. A formal baseline comparison with the overall in-center HD population of the same center was not part of the prespecified analysis, and comorbidity scores such as the Charlson index were not systematically available. In addition, the telemedicine component was introduced late in the observation period, patient-reported outcomes were not systematically collected, and the economic analysis did not include telemedicine implementation costs. Nevertheless, the inclusion of all patients treated over an extended observation period provides robust real-world evidence in a field still dominated by short-term or highly selected experiences.

In summary, in a real-world public healthcare setting, telemedicine-supported A-HHD appears as a feasible and sustainable dialysis modality for elderly and clinically complex patients. Adequate dialysis delivery can be achieved through flexible, frequency-adjusted treatment schedules, supporting more inclusive and patient-centered models of care. By enabling organizational sustainability and reducing transportation-related resource use, this model aligns with emerging strategies for equitable, technology-enabled, and economically sustainable kidney care [6, 9–11].

## Data Availability

All data supporting the findings of this study are available upon request.
